# Correlation of molecular and conventional methods of diagnosis in tuberculous lymphadenitis

**DOI:** 10.1186/1471-2334-14-S3-P27

**Published:** 2014-05-27

**Authors:** Bineeta Kashyap, Neha Goel, Rajneesh Avasthi, Vinod Arora, Iqbal R Kaur

**Affiliations:** 1 Department of Microbiology, University College of Medical Sciences & Guru Teg Bahadur Hospital, Delhi, India; 2 Department of Medicine, University College of Medical Sciences & Guru Teg Bahadur Hospital, Delhi, India; 3 Department of Pathology, University College of Medical Sciences & Guru Teg Bahadur Hospital, Delhi, India

## Background

Incidence of tuberculous lymphadenitis, accounting for 30 – 40% of extra-pulmonary tuberculosis, has increased in parallel with increase in the incidence of tuberculosis worldwide. The diagnosis becomes more challenging when clinical presentation is suggestive but bacteriological proof is lacking.

## Methods

Sixty lymph node aspirates from suspected cases of tuberculous lymphadenitis were examined for *Mycobacterium tuberculosis* by Ziehl neelsen (ZN) staining, culture on a Lowenstein Jensen medium, and cytological examination for findings suggestive of tubercular lymphadenitis. DNA from all the samples was amplified by conventional PCR targeting 123 bp and 240 bp fragments of *IS6110* and *MPT64* genes respectively and RealTime PCR targeting *MPT64* gene only.

## Results

Histopathology, ZN staining, and culture gave positivity in 25, 20 and 16 cases respectively; out of which 6 (10%) were diagnosed by positivity in all the three conventional techniques, whereas 36 (60%) were positive by any of these three techniques. Diagnosis could be made by conventional PCR in 43 cases when compared to 45 by the RealTime PCR method (7.2 vs 7.5%) while targeting the *MPT 64* gene in PCR could diagnose 45 cases unlike *IS6110* gene which could help in diagnosis of 42 cases (7.5 vs 7%). Whereas PCR gave 13 positivities which were negative by any of the conventional methods, it was negative in 2 samples that were positive on cytology only.

## Conclusion

Though conventional diagnosis remains the method of choice in tuberculous lymphadenitis alternative diagnostic method such as PCR which are more rapid and reliable looks promising and help well manage patients.

